# In vitro propagation of three mosaic disease resistant cassava cultivars

**DOI:** 10.1186/s12896-020-00645-8

**Published:** 2020-09-29

**Authors:** Amitchihoué Franck Sessou, Jane W. Kahia, Jerome Anani Houngue, Elijah Miinda Ateka, Colombe Dadjo, Corneille Ahanhanzo

**Affiliations:** 1 Department of Genetics and Biotechnology, Faculty of Sciences and Techniques, University of Abomey-Calavi, Abomey-Calavi, Benin; 2 Institute of Basic Sciences, Technology and Innovation, Pan African University, P. O. Box 62000-00200, Nairobi, Kenya; 3 Coffee Research Institute, P.O. Box 4, Ruiru, Kenya; 4 Department of Horticulture, Jomo Kenyatta University of Agriculture and Technology, P.O. Box 62000-00200, Nairobi, Kenya

**Keywords:** Cassava mosaic disease, SSR and SCAR markers, In-vitro propagation nodal explant, Genetic conformity

## Abstract

**Background:**

Cassava is a staple food for over 800 million people globally providing a cheap source of carbohydrate. However, the cultivation of cassava in the country is facing to viral diseases, particularly cassava mosaic disease (CMD) which can cause up to 95% yield losses. With aim to supply farmers demand for clean planting materials, there is need to accelerate the production of the elite cultivars by use of tissue culture in order to cope with the demand.

**Methods:**

Nodal explants harvested from the greenhouse grown plants were sterilised using different concentrations of a commercial bleach JIK (3.85% NaOCl) and varying time intervals. Microshoots induction was evaluated using thidiazuron (TDZ), benzyl amino purine (BAP), and kinetin. Rooting was evaluated using different auxins (Naphthalene acetic acid NAA and Indole-3-butyricacid IBA). PCR-based SSR and SCAR markers were used to verify the presence of *CMD2* gene in the regenerated plantlets.

**Results:**

The highest level of sterility in explants (90%) was obtained when 20% Jik was used for 15 min. The best cytokinin for microshoots regeneration was found to be kinetin with optimum concentrations of 5, 10 and 20 μM for Agric-rouge, Atinwewe, and Agblehoundo respectively. Medium without growth regulators was the best for rooting the three cultivars. A survival rate of 100, 98, and 98% was recorded in the greenhouse for Agric-rouge, Atinwewe, and Agblehoundo respectively and the plantlets appeared to be morphologically normal. The SSR and SCAR analysis of micropropagated plants showed a profile similar to that of the mother plants indicating that the regenerated plantlets retained the *CMD2* gene after passing through in vitro culture, as expected with micropropagation.

**Conclusion:**

The nodal explants was established to be 20% of Jik (3.85% NaOCl) with an exposure time of 15 min. Kinetin was proved to be the best cytokinins for microshoot formation with the optimum concentration of 5, 10 and 20 μM for Agric-rouge, Atinwewe, and Agblehoundo respectively. The protocol developed during this study will be useful for mass propagation of the elite cassava cultivars.

## Background

Cassava (*Manihot esculenta* Crantz) is a perennial woody shrub which belongs to the family *Euphorbiaceae*. It is a staple food for over 800 million people globally providing a cheap source of carbohydrates [1]. Cassava was introduced in West Africa from South America in 1558 (sixteenth century) and into East Africa in the eighteenth century [2, 3].

Cassava is the second staple food crop after maize in Benin Republic with 55% of farmers cultivating it [4]. However, the crop has major production constraints ranging from biotic to abiotic threats. According to Agre et al. [5] the most reported diseases among farmers (68%) were viral infections. These diseases have caused the yield to be much lower (15.55 t per hectare per year) than the potential global yield of 90 t per hectare per year [6]. This is attributed to a large extent to the devastating effects of cassava mosaic disease (CMD) causing a heavy yield loss in cassava [7, 8]. CMD is caused by cassava mosaic begomoviruses (CMBs). To date, the most successful approach used to control these viruses has been the introgression of *CMD1* which is polygenic recessive resistance locus, from wild cassava to cassava cultivars, or the use of natural resistant cassava cultivars from West Africa that contain *CMD2* which is dominant monogenic resistance locus [9]. The control measures against CMD include rogueing (removal of infected plants), the use of virus-free planting materials and resistant varieties [10]. Recent research efforts in Benin have led to the identification of *CMD2* resistant cultivars (Agric-rouge, Atinwewe, and Agblehoundo) [8]. The availability of these cultivars is a significant contribution towards the management of CMD causal viruses in Benin [8]. The propagation of these new cassava cultivars is by cuttings. This method is not only limiting in the numbers of planting materials but is also cumbersome, and labour intensive. Therefore, there is need to evaluate alternative propagation methods that are fast and tissue culture offers a feasible option.

Plant tissue culture as an important tool has been widely employed in area of agriculture, horticulture, forestry and plant breeding. It is an applied biotechnology used for mass propagation, virus elimination, secondary metabolite production and in-vitro cloning of plants [11–13]. Tissue culture depends on the effectiveness of the sterilization methods used on the explants prior to culture initiation. All the materials used in the plant tissue culture must be sterilized to kill the microorganisms that are present by using an appropriate sterilizing agents. Many protocols have been reported on surface sterilisation of cassava nodal explants from greenhouse. For instance, Abd-Alla et al. [14] reported effective sterilization of cassava nodes using Clorox (contains 5.25% NaOCl) at concentration of 20% for 15 min. Demeke et al. [15] sterilized cassava nodal explants from greenhouse by exposing them to 0.1% NaOCl with 1–3 drops of Tween-20 for 10 min, after initial soaking in 70% ethyl alcohol for 1 min while, Magaia, [16] reported the highest (87%) clean explants when nodal explants from greenhouse were exposed to 70% ethanol for 1 or 2 min followed by exposure to 0.05% HgCl2 for 2 min or 0.1% HgCl2 for 1 min. During the current study Jik (3.85% NaOCl) was used at different concentrations and intervals. Nodal culture is probably one of the safest methods of micropropagation because it has been shown to produce true-to-type plants. A range of cytokinins such as 6-benzylaminopurine (BAP), thidiazuron (TDZ), zeatin, and kinetin has been efficiently tested in cassava micropropagation to induced shoot regeneration. For instance, it has been reported that MS media supplemented with 10 mg/L BAP induced multiple-shoots with highest (25 shoots) mean number of shoots [17]. On the other hand, Mapayi et al. [18] reported that full MS [19] medium supplemented with NAA: 0.01 mg/L and BAP: 0.05 mg/L regenerated 100% plantlets. Kinetin also has been used at 0.75 mg/L to induce an average of 7.30 microshoots/ explant [15]. Auxins play a crucial role in rooting regenerated microshoots [11]. In cassava, rooting of in vitro derived microshoots has been reported by many authors. Mapayi et al. [18] reported that NAA (0.01–10 mg/L) as the most widely used and effective in cassava. Medina et al. [20] also reported that 0.54 mM NAA was most effective in stimulating root formation. Demeke et al. [15] used 0.5 mg/L NAA and reported the production of 6.14 roots within 4 weeks while Cacaï et al. [11] used 0.1 mg/L NAA and reported the production of 5.2 roots. On the other hand, Tadu, [21] reported that IAA at 0.02 mg/L and IBA at 0.04 mg/L were the best for rooting short maturing cassava genotypes and 0.06 mg/L IBA was the best for long maturing genotypes. It has been generally reported that MS medium without exogenous auxins has been proved to be best in cassava microshoots rooting [22, 23]. During the current study, IBA and NAA at different concentrations (0, 5, 10, 20 and 25 μM) were evaluated for their effectiveness to induce roots.

The plantlets derived from tissue culture may sometimes present a variation or lose *CMD2*- mediated resistance [24, 25]. The appropriate procedures for preventing its occurrence and the development of early detection methods are important factors for ensuring uniformity in the production of micropropagated plantlets. One of the early detection methods is the molecular detection based on the use of molecular markers, which are part of the genome, thus excludes both environmental effects and misidentifications. Microsatellite-based marker techniques such as Simple Sequence Repeat (SSR) and Sequence-Characterized Amplified Region (SCAR) markers have successfully been used in detection of *CMD2* resistant gene in cassava [8, 26]. The objectives of the current work was to establish a feasible in vitro protocol for propagating the three resistant cassava cultivars by determining the optimal sterilization technique for cassava nodal explants, then the effect of cytokinins (TDZ, kinetin, and BAP) on microshoots induction and the effect of auxins (NAA and IBA) concentrations on microshoots rooting, and also to assess the conformity of the regenerated plantlets using SSR and SCAR markers.

## Results

### Effect of JIK (3.85%NaOCl) on surface sterilization of cassava nodal explants

The results of sterilization of nodal explants from different cassava cultivars (S1 Table) are summarized in Table 1. The number of contaminated explants increased drastically from day 4 to day 12 following incubation on MS medium. After 12 days, explants of different cultivars sterilized using 20% Jik for 15 min gave the highest per cent (90%) clean explants while the lowest (13.33%) number of clean explants were obtained using 10% Jik for 15 min. Thus, sterilizing explants using 20% Jik for 15 min was more efficient for different cassava cultivars used.

**Table 1:** Effects of different concentrations of commercial bleach (Jik) on elimination of surface contamination of cassava nodal explants

Nb of explants	jik Concentration (%)	Exposure time (min)	Percent (%) clean explants		
			After 4 days	After 8 days	After 12 days
60	10	5	80	66.67	13.33
60	10	10	81.67	63.33	13.33
60	10	15	78.33	21.67	13.33
60	10	20	90	78.33	55
60	15	5	91.67	56.67	43.33
60	15	10	93.33	21.67	28.33
60	15	15	100	100	73.33
60	15	20	100	71.67	71.67
60	20	15	100	96.67	90
60	20	20	93.33	91.67	81.67
60	25	15	95	80	80
60	25	20	96.67	91.67	85

### Effect of cytokinins on microshoots formation

**Fig. 1:**
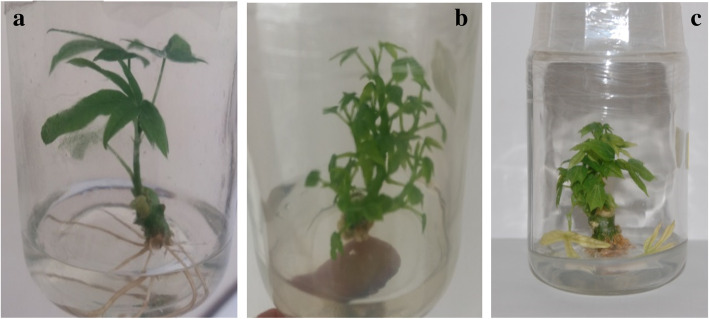
Microshoots regenerated from nodal explants **a**: single shoot on hormone free media; **b**: multiples shoots on medium supplemented with kinetin and **c** multiples shoots with callus at basal part on medium supplemented with BAP

The results of the effects of different cytokinins (Kinetin, BAP, and TDZ) are shown in Table 2. Generally, it was observed that 100% explants of all the cultivars evaluated sprouted within 2–3 days. Microshoots regeneration of the three cassava cultivars was influenced differently by the concentrations of the cytokinins used (S1 File). There was a significant difference (p < 0.001) in all parameters evaluated (number of shoots and microshoots length, as well as number of nodes) among all concentrations of BAP and kinetin but no difference was observed in the control and TDZ media. The result of the effects of cytokinins on microshoots formation in Agric-rouge are presented in Table 2. BAP at 10 μM gave the highest number of microshoots/explant (3.60 ± 0.03). While 5 μM kinetin induced the highest shoots length (6.32 ± 0.01). It was observed that increasing the concentration of both BAP and Kinetin beyond 10 μM significantly reduced the number of microshoots /explant and their lengths. However, TDZ (at all concentrations evaluated) did not affect the number of microshoots/explant. As for the cultivar Atinwewe (Table 2) BAP at 5 μM and 10 μM induced the highest number of microshoots/explant (2.84 ± 0.01 and 2.83 ± 0.01 respectively) while TDZ at 0.5 μM and 1 μM gave the highest mean length of 2.44 ± 0.01 cm and 2.33 ± 0.02 cm respectively. Kinetin at 20 μM induced the highest mean of 3.00 ± 0.89 microshoots per explant in the cultivar Agblehoundo (Table 2). However, this was not significantly different from the number (2.90 ± 1.00) of microshoots produced on the media supplemented with kinetin 10 μM. TDZ at 0.5 μM induced the highest microshoots mean length of 4.58 ± 0.05 cm. It was observed that the control and TDZ media produced single shoot while those cultured on BAP and kinetin media produced multiple shoots (Fig. 1) (S2 File).

**Table 2:** Effect of TDZ, BAP and Kinetin on microshoot formation in different cultivars after 3 weeks

Cytokinin conc. (μM)	Nb. of explants	Agric-rouge			Atinwewe			Agblehoundo		
		NME (M ± SE)	ML (M ± SE)	NNM (M ± SE)	NME (M ± SE)	ML (M ± SE)	NNM (M ± SE)	NME (M ± SE)	ML (M ± SE)	NNM (M ± SE)
**0**	30	1.00 ± 0.00^f^	4.67 ± 0.02^de^	3.18 ± 0.01^c^	1.00 ± 0.00^f^	1.84 ± 0.04^cd^	3.04 ± 0.03^cd^	1.00 ± 0.00^e^	2.20 ± 0.01^f^	3.04 ± 0.03^d^
**TDZ-0.1**	30	1.00 ± 0.00^f^	5.66 ± 0.01^b^	3.12 ± 0.02^c^	1.00 ± 0.00^f^	1.96 ± 0.02^c^	3.06 ± 0.07^c^	1.00 ± 0.00^e^	3.17 ± 0.01^e^	3.10 ± 0.01^d^
**TDZ-0.5**	30	1.00 ± 0.00^f^	5.15 ± 0.03^cd^	3.40 ± 0.02^bc^	1.00 ± 0.00 ^f^	2.44 ± 0.01a	3.35 ± 0.05^a^	1.00 ± 0.00^e^	4.58 ± 0.05^a^	3.62 ± 0.01^a^
**TDZ-1**	30	1.00 ± 0.00^f^	2.72 ± 0.32^f^	3.24 ± 0.02^c^	1.00 ± 0.00^f^	2.33 ± 0.02^ab^	2.67 ± 0.02^e^	1.00 ± 0.00^e^	3.23 ± 0.01^e^	2.68 ± 0.03^e^
**TDZ-1.5**	30	1.00 ± 0.00^f^	4.26 ± 0.04^e^	3.24 ± 0.28^c^	1.00 ± 0.00^f^	1.83 ± 0.01^d^	2.58 ± 0.01^e^	1.00 ± 0.00^e^	2.18 ± 0.01^f^	2.43 ± 0.01^f^
**BAP-5**	30	3.00 ± 0.06^c^	1.10 ± 0.03^g^	1.83 ± 0.01^d^	2.84 ± 0.01^a^	1.36 ± 0.06^e^	1.57 ± 0.01^f^	2.04 ± 0.03^c^	0.50 ± 0.01^g^	1.00 ± 0.00^h^
**BAP- 10**	30	3.60 ± 0.03^a^	0.65 ± 0.01^gh^	1.31 ± 0.04^e^	2.83 ± 0.01^a^	0.81 ± 0.02^f^	1.67 ± 0.02^f^	2.25 ± 0.01^b^	0.29 ± 0.01^h^	1.03 ± 0.03^h^
**BAP-20**	30	2.00 ± 0.12^e^	0.33 ± 0.01^h^	1.07 ± 0.07^e^	1.90 ± 0.02^d^	0.39 ± 0.01^g^	1.05 ± 0.03^g^	1.17 ± 0.02^d^	0.22 ± 0.02^h^	1.00 ± 0.00^h^
**Kin-5**	30	3.27 ± 0.01^b^	6.32 ± 0.01^a^	4.20 ± 0.05^a^	2.00 ± 0.02^c^	2.31 ± 0.01^b^	2.55 ± 0.01^e^	2.00 ± 0.44^c^	4.18 ± 0.01^b^	3.33 ± 0.02^b^
**Kin-10**	30	3.18 ± 0.01^bc^	5.60 ± 0.01^bc^	3.82 ± 0.33^ba^	2.32 ± 0.02^b^	2.27 ± 0.02^b^	3.25 ± 0.02^b^	2.90 ± 1.00^a^	3.95 ± 0.01^c^	3.22 ± 0.01^c^
**Kin-20**	30	2.55 ± 0.2^d^	3.08 ± 0.01^f^	2.28 ± 0.04 ^d^	1.39 ± 0.02^e^	1.94 ± 0.02^cd^	2.90 ± 0.01^d^	3.00 ± 0.89^a^	3.71 ± 0.01^d^	2.19 ± 0.02^g^

*Means followed by the same letter are not significantly different at *P* ≤ 0.05

***NME*** Number of microshoots per explant**,** ***ML*** Microshoot length (cm), ***NNM*** Number of nodes per microshoot, ***M*** Mean, ***SE*** Standard error, ***TDZ*** Thidiazuron**,** *BAP* Benzyaminopurine, *Kin* Kinetin

### Effect of auxins on roots formations

For all the three cultivars, the number of roots and roots length were significantly (*p* ≤ 0.001) affected by both auxins tested (S3 File). Medium without growth regulators was found to be the best in term of roots induction (100%) while frequencies of roots induction with media supplemented with NAA and IBA were 59 and 85% respectively. However, IBA gave the highest mean number of roots in all cultivars. Roots induction in Agric-rouge microshoots regenerated from medium supplemented with kinetin, TDZ and BAP are shown in Table 3.

**Table 3:** Effect of auxins on rooting of microshoots regenerated from media supplemented with TDZ, kinetin, and BAP in cultivar Agric-rouge after 2 weeks

Auxins conc. (μM)	Nb. of microshoot	TDZ		Kinetin		BAP	
		Number of roots	Roots length (cm)	Number of roots	Roots length (cm)	Number of roots	Roots length (cm)
**0**	30	3.58 ± 0.02^b^	3.76 ± 0.05^a^	3.62 ± 0.02^d^	2.17 ± 0.02^b^	2.63 ± 0.0^h^	1.21 ± 0.03^c^
**IBA- 1**	30	1.21 ± 0.01^ef^	0.33 ± 0.02^f^	2.52 ± 0.03^e^	1.28 ± 0.01^e^	3.27 ± 0.02^f^	0.98 ± 0.03^d^
**IBA-5**	30	2.62 ± 0.03^c^	1.34 ± 0.02^c^	5.23 ± 0.03^c^	1.48 ± 0.03^d^	4.22 ± 0.02^d^	0.83 ± 0.02^e^
**IBA-10**	30	4.81 ± 0.02^a^	2.96 ± 0.03^b^	18.83 ± 0.02^a^	1.83 ± 0.02^c^	13.16 ± 0.03^a^	1.80 ± 0.02^b^
**IBA-20**	30	1.51 ± 0.02^e^	0.54 ± 0.02^d^	14.88 ± 0.02^b^	2.30 ± 0.03^a^	10.46 ± 0.02^b^	2.26 ± 0.03^a^
**NAA-1**	30	2.01 ± 0.03^d^	0.61 ± 0.02^d^	2.34 ± 0.52^e^	0.42 ± 0.03^g^	3.40 ± 0.02^e^	0.78 ± 0.05^e^
**NAA-5**	30	1.05 ± 0.05^f^	0.12 ± 0.02^f^	1.82 ± 0.02^f^	0.92 ± 0.02^f^	5.32 ± 0.03^c^	0.85 ± 0.03^e^
**NAA-10**	30	1.14 ± 0.04^f^	0.31 ± 0.02^e^	1.76 ± 0.03^f^	0.39 ± 0.10^g^	2.66 ± 0.03^h^	0.42 ± 0.04^g^
**NAA-20**	30	1.24 ± 0.34^ef^	0.15 ± 0.04^f^	4.03 ± 0.04^d^	1.34 ± 0.02^e^	2.85 ± 0.04^g^	0.58 ± 0.07^f^

*Means followed by the same letter are not significantly different at *P* ≤ 0.05

When the microshoots regenerating from media supplemented with kinetin were subcultured on MS media supplemented with IBA, they gave the highest (18.83 ± 0.02) mean number of roots on IBA 10 μM. Subculturing microshoots regenerated from a media supplemented with TDZ on hormoneless media gave the highest (3.76 ± 0.05 cm) roots length. It was observed that increasing the concentration of IBA from 10 to 20 μM significantly reduced the mean number of roots in microshoots derived from the media supplemented with the three cytokinins. The results of induction of roots from Atinwewe microshoots regenerated from medium supplemented with kinetin, TDZ and BAP are shown in Table 4.

**Table 4:** Effect of auxins on rooting of microshoots regenerated from media supplemented with TDZ, kinetin, and BAP in cultivar Atinwewe after 2 weeks

Auxins conc. (μM	Nb. of microshoot	TDZ		Kinetin		BAP	
		Number of roots	Roots length (cm)	Number of roots	Roots length (cm)	Number of roots	Roots length (cm)
**0**	30	9.03 ± 0.08^b^	4.65 ± 0.02^a^	4.87 ± 0.02^c^	2.16 ± 0.02^c^	3.88 ± 0.02^d^	2.56 ± 0.03^a^
**IBA- 1**	30	2.27 ± 0.03^g^	1.42 ± 0.03^e^	2.23 ± 0.02^h^	1.28 ± 0.02^f^	4.05 ± 0.05^c^	1.25 ± 0.02^c^
**IBA-5**	30	8.22 ± 0.03^c^	1.91 ± 0.02^c^	4.27 ± 0.02^d^	2.03 ± 0.05^d^	7.21 ± 0.03^a^	0.97 ± 0.02^e^
**IBA-10**	30	1.75 ± 0.02^h^	1.54 ± 0.02^d^	8.06 ± 0.12^b^	2.66 ± 0.03^a^	3.56 ± 0.02^e^	1.09 ± 0.03^d^
**IBA-20**	30	17.26 ± 0.03^a^	2.69 ± 0.06^b^	9.24 ± 0.06^a^	2.34 ± 0.01^b^	3.82 ± 0.02^d^	1.06 ± 0.03^d^
**NAA-1**	30	2.55 ± 0.05^f^	0.61 ± 0.03^f^	2.54 ± 0.05^g^	1.68 ± 0.02^e^	2.75 ± 0.02^g^	0.55 ± 0.02^g^
**NAA-5**	30	4.84 ± 0.01^d^	0.42 ± 0.03^g^	3.65 ± 0.02^e^	1.51 ± 0.02^e^	2.66 ± 0.03^g^	0.65 ± 0.02^f^
**NAA-10**	30	3.81 ± 0.02^e^	1.45 ± 0.02^e^	2.34 ± 0.02^h^	1.18 ± 0.02^g^	3.45 ± 0.05^f^	0.93 ± 0.03^e^
**NAA-20**	30	2.21 ± 0.02^g^	0.44 ± 0.02^g^	3.03 ± 0.03^f^	1.24 ± 0.02^fg^	4.75 ± 0.02^b^	1.38 ± 0.02^b^

*Means followed by the same letter are not significantly different at *P* ≤ 0.05

When microshoots regenerated from media supplemented with TDZ were subcultured on MS media supplemented with IBA, they gave the highest (17.26 ± 0.03) mean number of roots on IBA 20 μM and the control gave the highest (4.65 ± 0.02 cm) mean length. It was observed that increasing the concentration of IBA from 1 to 20 μM increased the mean number of roots in microshoots derived from the media supplemented with TDZ and kinetin. However, increasing the concentration of NAA from 5 to 20 μM significantly inhibited the roots formation and reduced the mean number of roots and their length. Agblehoundo microshoots regenerated from media supplemented with kinetin gave the highest (16.38 ± 0.02 roots per shoot) mean number of roots when subcultured on media with IBA 20 μM (Table 5).

**Table 5:** Effect of auxins on rooting microshoots regenerated from media supplemented with TDZ, kinetin, and BAP in cultivar Agblehoundo after 2 weeks

Auxins conc. (μM)	Nb. of microshoot	TDZ		Kinetin		BAP	
		Number of roots	Roots length (cm)	Number of roots	Roots length (cm)	Number of roots	Roots length (cm)
**0**	30	2.77 ± 0.02^d^	1.82 ± 0.02^c^	4.26 ± 0.01^c^	0.91 ± 0.10^c^	2.43 ± 0.03^g^	0.42 ± 0.03^d^
**IBA- 1**	30	2.57 ± 0.05^e^	1.86 ± 0.03^c^	3.55 ± 0.02^f^	1.09 ± 0.10^b^	2.91 ± 0.03^e^	0.30 ± 0.04^e^
**IBA-5**	30	3.00 ± 0.09^c^	2.30 ± 0.03^a^	5.97 ± 0.08^b^	0.76 ± 0.01^cd^	3.19 ± 0.03^d^	0.39 ± 0.02^d^
**IBA-10**	30	4.75 ± 0.05^b^	2.14 ± 0.03^b^	3.43 ± 0.01^e^	0.54 ± 0.02^e^	4.14 ± 0.03^c^	0.75 ± 0.02^b^
**IBA-20**	30	8.75 ± 0.07^a^	1.55 ± 0.02^d^	16.38 ± 0.02^a^	1.94 ± 0.03^a^	7.05 ± 0.05^a^	0.99 ± 0.04^a^
**NAA-1**	30	2.01 ± 0.023^f^	1.41 ± 0.02^e^	2.17 ± 0.02^h^	0.53 ± 0.04^e^	2.60 ± 0.03^f^	0.78 ± 0.03^b^
**NAA-5**	30	2.02 ± 0.02^f^	1.26 ± 0.02^f^	2.35 ± 0.03^g^	0.64 ± 0.06^de^	4.46 ± 0.02^b^	0.60 ± 0.03^c^
**NAA-10**	30	1.76 ± 0.03^g^	1.15 ± 0.05^g^	3.75 ± 0.06^d^	0.61 ± 0.08^de^	2.90 ± 0.02^e^	0.38 ± 0.03^de^
**NAA-20**	30	1.44 ± 0.01^h^	0.55 ± 0.02^h^	2.25 ± 0.02^gh^	0.55 ± 0.02^e^	2.34 ± 0.03^h^	0.43 ± 0.04^d^

*Means followed by the same letter are not significantly different at *P* ≤ 0.05

**Fig. 2:**
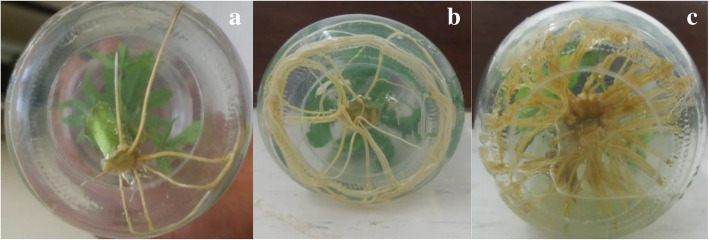
In vitro rooting: **a**- Roots formation after 2 weeks; **b**- Plantlets rooting on plain media after 4 weeks; **c**-Roots of plantlets on IBA after 4 weeks

Subculturing microshoots regenerated from TDZ supplemented media on IBA 5 μM gave the highest (2.30 ± 0.03 cm) roots length. It was observed that increasing the concentration of IBA from 1 to 20 μM significantly increased the mean number of roots in microshoots derived from the media supplemented with the three cytokinins. In the converse, increasing the concentration of NAA from 5 to 20 μM significantly inhibited the roots formation. The roots obtained in shoots subcultured on the control were few and more elongated while the roots derived from microshoots subcultured on IBA media were many and shorter (Fig. 2).

### Acclimatization

**Fig. 3:**
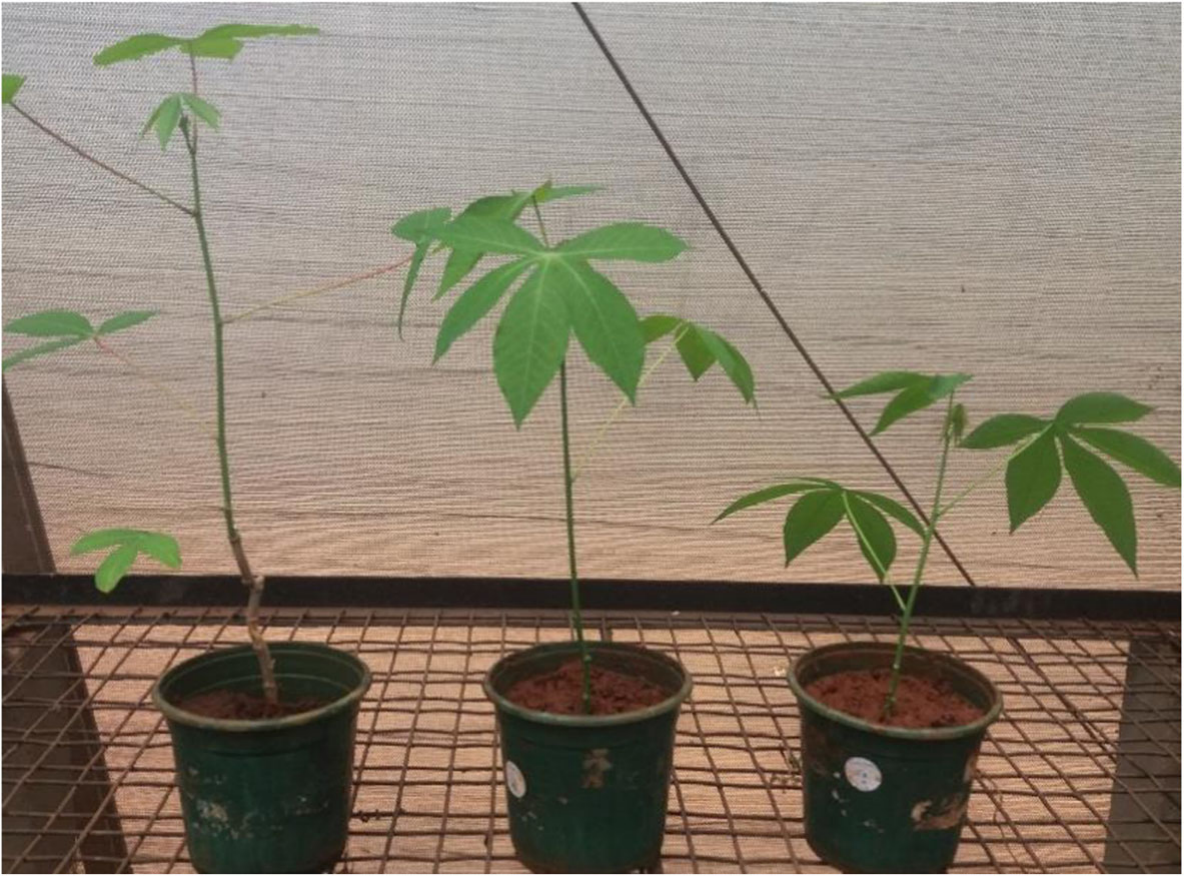
Plantlets after one month in the greenhouse

The survival rate of the in vitro plantlets in the greenhouse was in the range of 98–100%. No morphological differences between regenerated and mother plants were observed in Fig. 3.

### Molecular assessment of similarity in plants derived from micropropagation

**Fig. 4:**
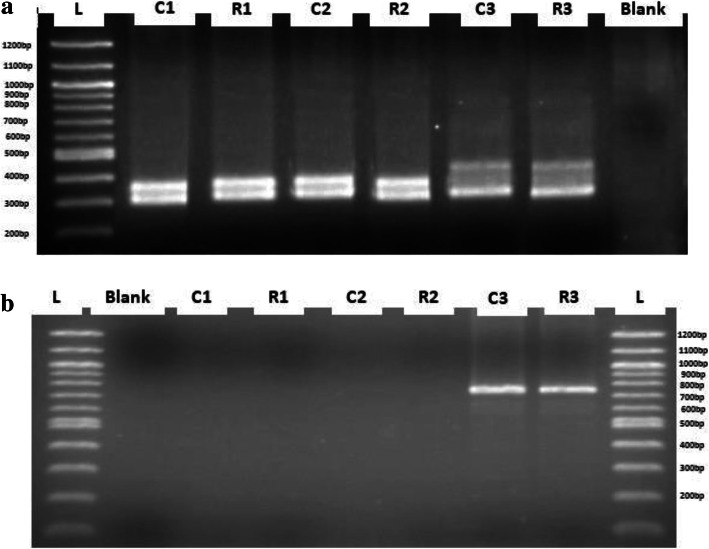
**a-** SSR banding patterns with primers NS169 and **b-** SCAR banding patterns with primers RME1 in both micropropagated and greenhouse-grown mother plants Legend: C1: Mother plant of Agric-rouge (positive control); C2: Mother plant of Atinwewe (positive control); C3: Mother plant of Agblehoundo (positive control); R1: Regenerated plant of Agric-rouge; R2: Regenerated plant of Atinwewe; R3: Regenerated plant of Agblehoundo; Blank (master mix without DNA): Negative control; L: Loader. The primer NS169 amplified 319 base pairs of CMD2 gene sequence and the primer RME1 amplified 700 base pairs of CMD2 gene sequence

At the end of the hardening, an initial molecular analysis was conducted on the regenerated plants and the donor plants in order to confirm the similarity of micropropagated *CMD2* resistant cassava cultivars maintained in culture over a period of 6 months. Twenty base pairs of SSR and twenty-one base pairs of SCAR primers were used for PCR analysis. The SSR primer generated a total of 319 bp amplification fragments (*CMD2* resistant gene) while SCAR primer generated a total of 700 bp amplification fragments of the *CMD2* resistant gene. All of the primers produced monomorphic amplification patterns in the regenerated plants, and no differences were found in the amplification pattern among regenerated plants and donor plants (Fig. 4). One regenerated plant was tested with its donor plant as positive control for each cultivar with three repetitions.

## Discussion

The current study was conducted with the aim of optimizing the sterilization of nodal explants and the in vitro propagation of three mosaic disease resistant cassava cultivars from Benin. Nodal explants are the most difficult to sterilize during tissue culture and despite using high concentrations of sterilizing agents such as Parazone, Domestos, and Jik, they have been found to be ineffective resulting in many cases 100% contamination [27]. During the current study, exposing the nodal explants to 20% Jik (3.85% NaOCl) for 15 min gave the highest (90%) clean explants. These results concur with those of Maruthi et al. [28] who reported high percent (80–90%) clean explants when a commercial bleach (5% pure sodium hypochlorite) was used on some cassava cultivars. It also corroborates with the results of Waweru et al. [29] who reported that the highest proportion (92%) of clean explants was obtained when the *Cyphomandra betacea* nodal explants were exposed to 15% Jik for 20 min. Magaia, [16] while working on cassava nodal explants reported high (87%) numbers of clean explants when the explants were exposed twice to HgCl_2_ solution: 0.05% HgCl_2_ for 2 min followed by exposure to 0.1% HgCl_2_ for 1 min. However, HgCl_2_ solution is highly toxic and it is therefore recommended for frequent use in sterilization procedure.

Growth regulators especially cytokinins are one of the most important factors affecting the regeneration of microshoots [30–33]. Kinetin, BAP, TDZ and Zeatin have been used in cassava micropropagation [17, 34–36]. However, the most commonly used cytokinin to induce shoots in cassava is either BAP alone or in combination with NAA [11, 34, 37]. Results in the current study showed that the regeneration of microshoots from cassava nodal explants in the different cultivars depends on the type of cytokinins and the concentration. BAP was found to be the best cytokinins for microshoot regeneration in Agric-rouge and Atinwewe while kinetin was the best in Agblehoundo. It was also observed that the response depended on the concentration of the cytokinin for each cultivar. The results in Agblehoundo concur with those of Konan et al. [17] and Faye et al. [34] who found that kinetin gave better results than BAP in regenerating cassava microshoots from nodal explants. Kinetin has also been found to be superior than other cytokinins in *Tacca leontopetaloides* [38]. The results of the current study are contrary to those of Opabode et al. [39] who reported that BAP alone was best in cassava shoots induction. The combination of two cytokinins has also been found to be effective on some varieties of cassava. For example, Sukmadjaja and Widhiastuti [40] reported that the highest number of shoots from three elite cassava cultivars were obtained on media supplemented with a combination of BAP and TDZ. While working on two Ethiopian varieties of cassava, Demeke et al. [15] reported varietal differences in shoots formation when nodal explants were cultured on MS medium supplemented with BAP and kinetin. During the present study, it was observed that the multiple shoots regenerated from media supplemented with BAP were stunted compared to those obtained with kinetin. Onwubiku, [41] made similar observation and he reported that BAP considerably inhibited the performance (microshoots length, number of nodes, number of leaves) of two cassava varieties and Kirika et al. [42] also made the same observation while working with *Erythrina abyssinica*. In another study, combining BAP and NAA gave the best shoot elongation in some cassava varieties [43]. From the results of the current study, it can be deduced that kinetin would be a preferred cytokinin compared to BAP for inducing microshoots in the new cultivars since it produced multiple shoots that were elongated which could be easily subdivided for further multiplication.

Auxins are important factors involved in rooting because they promote adventitious roots formation in the vast majority of species [44]. During the current study, IBA was found to be better than NAA in rooting the microshoots from the 3 elite cassava cultivars. The results of this study are similar to those of Kabir et al. [35] who reported that cassava microshoots rooted well in MS media supplemented with IBA compared to NAA and IAA. The effectiveness of IBA for rooting over other auxins has also been reported by Naranjo and Fallas [45] in cassava. Similar observation was made in many other in vitro cultured plants. For instance, Sadeghi et al. [46] achieved 100% in vitro rooting of *Prunus empyrean* in MS medium with IBA and Singh et al. [47] reported IBA as the best auxin for rooting in *Santalum album*. The results of the study being reported are contrary to the observations made by Shiji et al. [43] and Opabode [36] who found NAA to be the best for rooting in cassava. A possible explanation for these differences could be the genetic makeup of the cultivars evaluated. It was also observed that 100% rooting occurred in microshoots cultured in the media devoid of any growth regulator. Similar observation was made by Faye et al. [34] and Yandia et al. [23] while they were working on various cassava cultivars. Rooting in medium without growth regulators have been reported in *Yucca glauca* [48] and *Gentiana dinarica Beck* plant [49]. A possible explanation could be that there is high level of endogenous auxins.

The SSR and SCAR analysis of micropropagated plants of cassava (Agric-rouge, Atinwewe, and Agblehoundo) showed a profile similar to that of the mother plants indicating that no variation had occurred in vitro. Through PCR amplification, SSR primer generated 319 bp amplicons which is a portion of the *CMD2* resistance gene carried by the three local cultivars while a DNA fragment of 700 bp size generated by SCAR generated 700 bp is carried by the local cultivar Agblehoundo alone. This result concurs with the work reported by Houngue et al. [8] where they detected the *CMD2* gene using the same primers as those used in this study. It is generally known that plantlets regenerated through nodal culture have lower risk of genetic instability. The present study provides the information on the conformity of micropropagated *CMD2* resistant cassava cultivars with mother plants using SSR and SCAR analysis. Micropropagation has an advantage over somatic embryogenesis in that it is thought to reduce the potential for undesirable variants among the regenerated plants, whereas in somatic embryogenesis the risk of genetic instability is high. For instance, Beyene et al. [25] reported that using somatic embryogenesis led to 100% loss of resistance to geminivirus pathogens of the regenerated *CMD2* plants. However, the plantlets were otherwise phenotypically indistinguishable from the CMD-resistant mother plants from which they were derived. Similarly, Chauhan et al. [50] showed that multiple morphogenic culture systems cause loss of resistance to cassava mosaic disease. In their study, they found that 25–36% and 5–10% of regenerated plant lines lost resistance to CMD respectively.

## Conclusions

A simple two-step regeneration method for propagating the new cassava cultivars was developed. The optimum Jik (3.85% NaOCl) concentration for sterilization of nodal explants was established to be 20% and an exposure time of 15 min. Kinetin proved to be the best cytokinins for microshoots formation with the optimum concentration of 5, 10 and 20 μM for Agric-rouge, Atinwewe, and Agblehoundo respectively. Medium without growth regulators was best for rooting the regenerated microshoots in all the 3 cultivars. Furthermore, SSR and SCAR primers confirmed the presence of the *CMD2* gene in regenerated plants through nodal culture similar to the mother plants. The three resistant cultivars used in this study are implicated in CMD management in West and Central Africa through West Afican Virus Epidemiology programme. The developed protocol will go a long way in providing farmers with the much-needed resistant cassava planting materials of the new cultivars for controlling the disease.

## Methods

### Plant materials

Cassava cuttings of the three *CMD2* resistant cultivars (Agric-rouge, Atinwewe, and Agblehoundo) were collected from University of Abomey-Calavi in Central Laboratory of Biotechnology and Plant breeding Gemoplasm in Benin and transported to Coffee Research Institute (CRI) in Ruiru-Kenya where the tissue culture studies were carried out. The cuttings were certified by ‘Plant Protection Organization of Benin’ on N° 0054994/19/SPVCP/PCP/AE-B (S4 File) before sent to the Coffee Research Institute (CRI) in Kenya. The 20-cm-long cuttings were planted as four to five stems in 10-L boat filled with sterile soil/manure mixture (1,1 v/v) (S5 File). The boat were irrigated to field capacity once per day until sprouting, and twice per week thereafter; cuttings were grown in a greenhouse maintained at 28 °C, with relative humidity > 60%, and natural lighting with an approximate light/dark cycle of 12/12 h at Coffee Research Institute (CRI) in Kenya**.**

### Explants sterilization

Nodal explants from 4 weeks old stem cuttings were harvested from each of three cassava cultivars (Agric-rouge, Atinwewe, and Agblehoundo) and transported from the greenhouse to the laboratory in a beaker containing tap water. Once in the laboratory, they were cleaned with cotton wool contained liquid soap to remove any surface debris and rinsed with tap water. Twenty nodal explants from different cassava cultivars were then sterilized in different treatment condition (Jik concentration and Exposure time) under the lamina flow hood using 10, 15, 20, and 25% v/v commercial bleach Jik (3.85% NaOCl) for 5, 10, 15, and 20 min. In total, twelve treatment conditions have been used. After exposure to the sterilant, the explants were rinsed two times in sterile distilled water and thereafter quickly (30 s) immersed in 70% (v/v) ethanol and finally rinsing four times in sterile distilled water. The nodal explants were trimmed and cultured individually in test tubes (15 cm by 3 cm) containing hormone free MS media. They were then incubated in a growth room maintained at a temperature regime of 25 ± 2 °C provided by with cool white fluorescent light intensity of 33 μmol. m^− 2^.s^− 1^ and 16 h photoperiod. Data on the percent clean explants were collected after 4, 8, and 12 days. This was calculated as total number of contaminated explants / total number of explants × 100.

### Microshoots induction and culture conditions

Nodal explants from plantlets of different cassava cultivars initially obtained were cultured on MS medium [19] basal salts supplemented with 3% (w/v) sucrose, 100 mg/l myo-inositol, BAP, kinetin evaluated at 5 μM, 10 μM, and 20 μM and TDZ at 0.1 μM, 0.5 μM, 1 μM, and 1.5 μM in separate experiments. The control was devoid of hormones. In total, thirty nodal explants of each cultivar were cultured on eleven MS media different by the concentration of cytokinins. The pH of the media was adjusted to 5.8 using 0.1 M HCl or 0.1 M NaOH, and the media was gelled with 0.3% phytagel. The media were dispensed in 20 mL aliquots into culture vessels and then autoclaved at 1.06 kg•cm-2 and 121 °C for 15 min. The number of microshoots/ explant, microshoots length, and number of nodes /microshoot were scored in each shooting condition.

### Roots induction and culture condition

Rooting of the microshoots was evaluated using half-strength MS media supplemented with 2% (w/v) sucrose, 100 mg/l myo-inositol, NAA and IBA evaluated at 1, 5, 10 and 20 μM in separate experiments. The control was devoid of hormone. In total, thirty microshoots of each cultivar regenerated from MS media supplemented with cytokinins were subcultured on ten half-strength MS media different by the concentration of auxins with pH = 5.8. The number of roots and roots length were scored in each rooting condition.

### Plantlets establishment in greenhouse

**Fig. 5:**
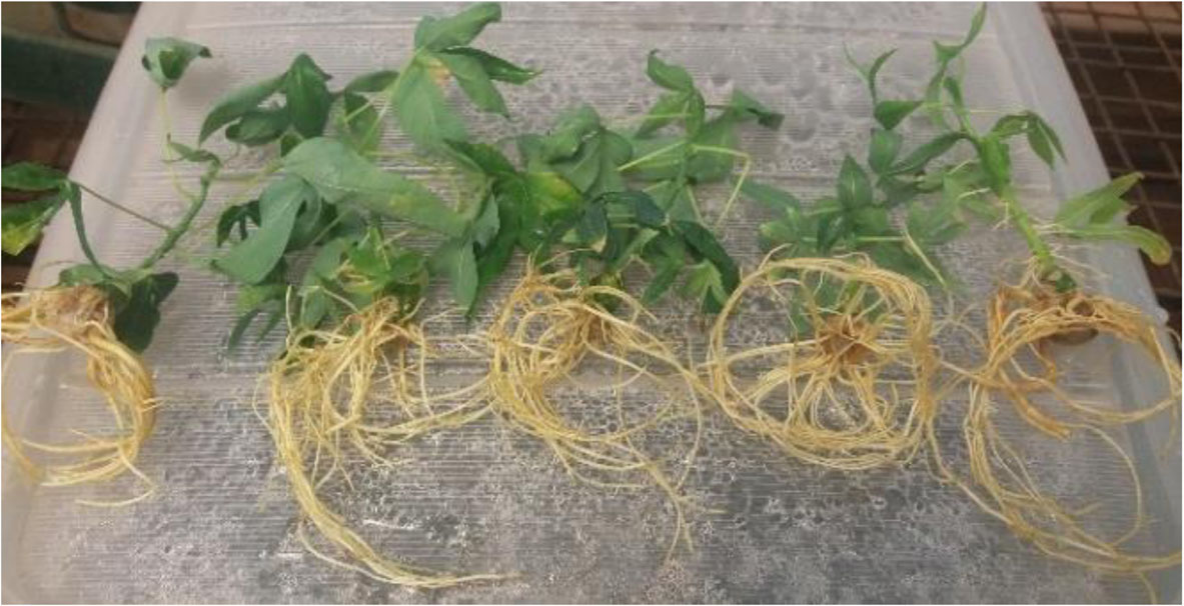
Plantlets from the lab after washing off the agar

**Fig. 6:**
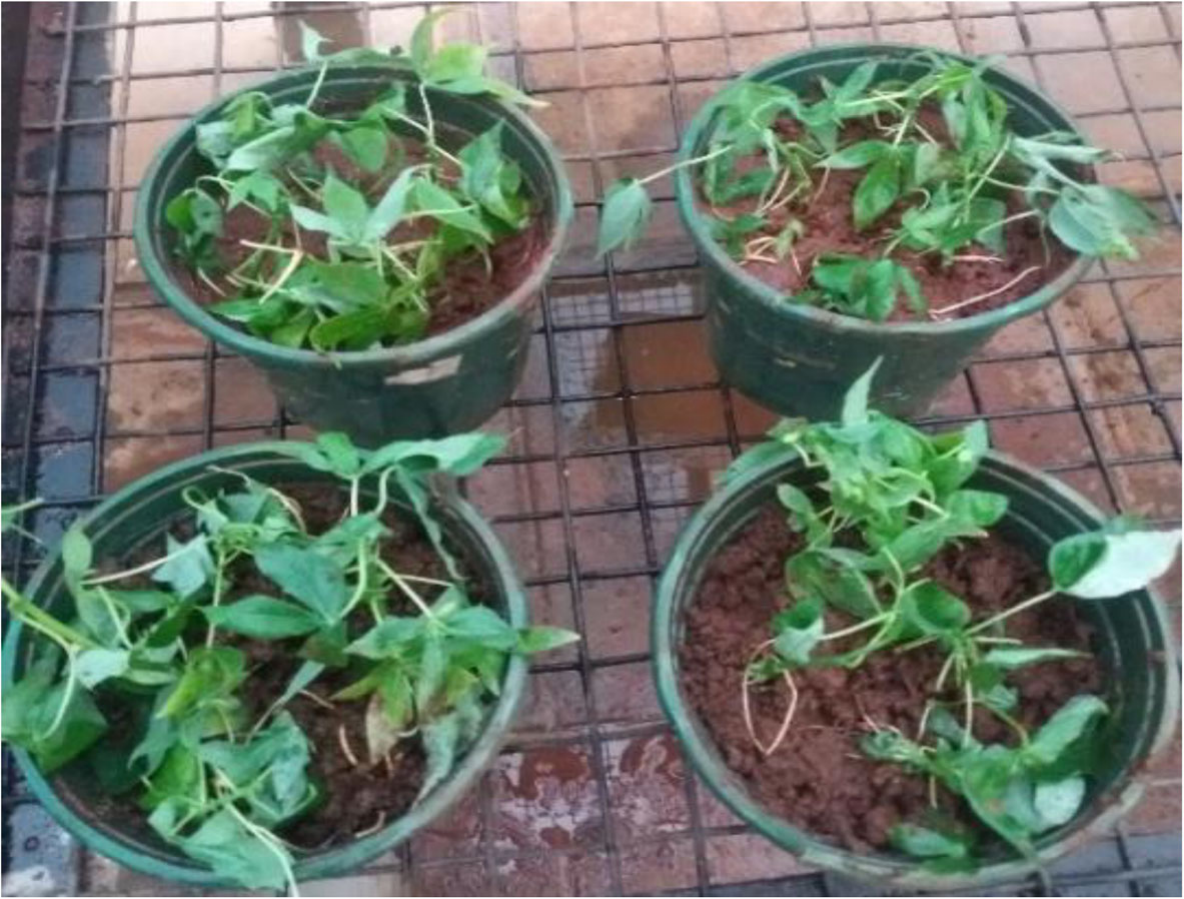
Plantlets transplanted in plastic pots filled with soil mixture (soil: sand: manure in the ratio of 3:2:1)

**Fig. 7:**
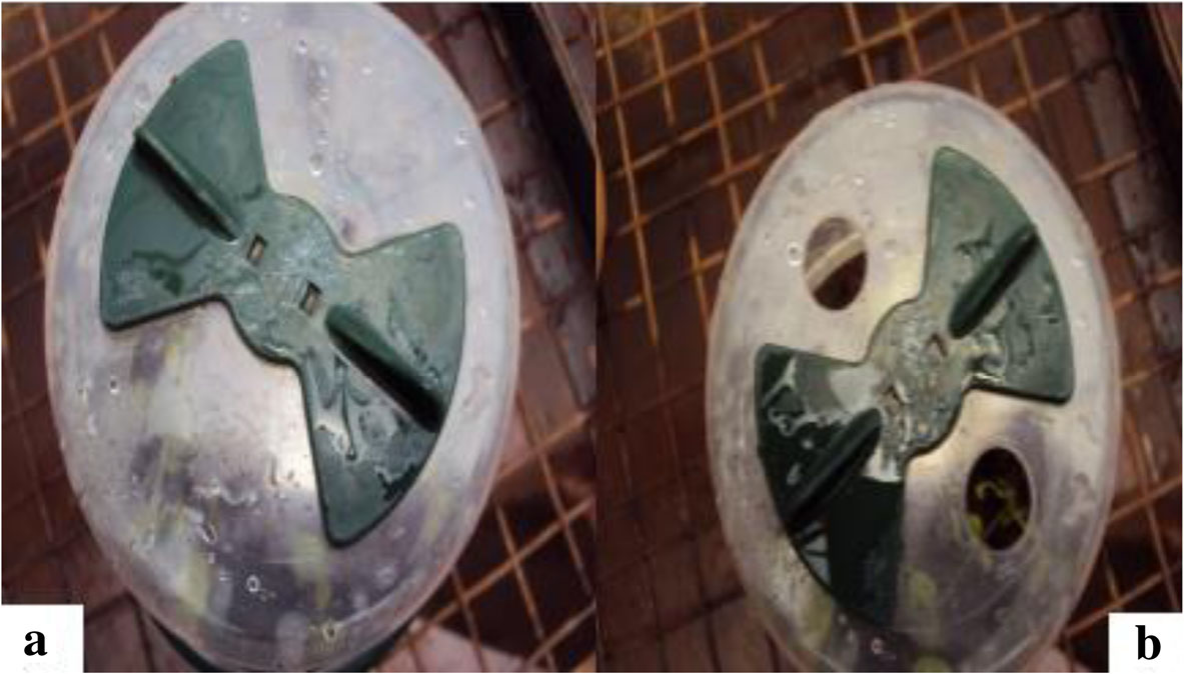
**a** - Covered weaning pots; **b** - Partially opened pots for reduction of humidity

The regenerated plantlets with well-developed roots were carefully removed from the culture tubes washed with tap water to remove agar (Fig. 5). They were then dipped in 2% fungicide (green copper) for 1 h. They were then placed in plastic pots filled with substrate composed of soil: sand: manure in the ratio of 3:2:1 (Fig. 6). The containers were covered to maintain high relative humidity. The humidity was reduced gradually by opening the top of the pots after 2 weeks (Fig. 7) (S6 File).

### Assessment of the presence CMD2 gene in plantlets

The molecular analysis work was done in the Molecular Biology and Biotechnology laboratories of CRI and Pan African University of Basic Sciences, Technology and Innovation (PAUSTI), Kenya.

### DNA extraction and quantification

Young leaves were picked from cassava plants in greenhouse, both mother plants and acclimatized plantlets, and DNA was extracted from the plantlets and the mother plants according to the method described by Diniz et al. [51]. DNA quality and quantity were determined with Genova Spectrophotometer (Model 7415 Nano, Vacutec, South Africa) and quality was also assessed on 1% (w/v) agarose gel. The extracted DNA samples were stored at − 20 °C for SSR and SCAR analysis.

### Polymerase chain reaction (PCR) for scoring *CMD2* resistant gene

During the current study, PCR-based SSR and SCAR markers as described by Houngue et al. [8] were used to verify the presence of *CMD2* gene in the regenerated plantlets. The mother plants were used as the positive control. The characteristics of the primers used are shown in Table 6. The SSR and SCAR analysis were performed as described by Omingo et al. [52]. DNA samples were diluted to10 ng/μl for SSR and SCAR analysis. A total of 100 ng of each DNA sample was used in PCR reactions. A reaction mix was prepared to include: 2.5 μl of buffer (10 x), 2.5 μl of MgCl2 (25 mM), 3.5 μl of dNTPs (500 μM), 1 μl of SSR (10 μM) reverse primer and 1 μl of forward primer, 0.2 μl of Taq polymerase 5u / μl. The 25 μl PCR volume was incubated in a thermocycler (Model FFG02HSD, made in UK) set for the following amplification conditions: One cycle at 95 °C for 5 min, 35 cycles of denaturation at 94 °C for 1 min, annealing at 55 °C for one and half minutes, extension at 72 °C for 10 min and was held 4 °C. The amplified products were electrophoresed in 2.3% agarose gel and then visualized in a UV trans-illuminator (Model M-26, Upland, CA 91786 U.S.A) after staining in ethidium bromide solution.

**Table 6:** Specific SSR and SCAR primers used for detection of CMD2 resistant gene in cassava mother plants and regenerated plantlets

Primer code	Marker system	Forward primer sequence	Reverse primer sequence	Expected sequence length (bp)	Annealing Temperature (°C)
NS169	SSR	GTGCGAAATGGAAATCAATG	GCCTTCTCAGCATATGGAGC	319	55
RME1	SCAR	AGAAGAGGGTAGGAGTTATGT	ATGTTAATGTAATGAAAGAGC	700	55

### Scoring and analysis of bands

Amplified DNA fragments were run on agarose gel to score for the presence (1) or absence (0) of bands (Resistance gene) in the regenerated plantlets compared with mother plants. The reactions were repeated at least twice, and only distinct, reproducible, polymorphic and well-resolved bands across all runs were considered for analysis.

### Experimental design and data analysis

All experiments on both shoot and root induction were laid out in completely randomized design (CRD) with 10 replicates per treatment and the experiment repeated three times. The data was subjected to one-way analysis of variance and the significant differences between treatments means were assed using MINITAB version 19 software and Tukey analysis at 5% level were performed to assess difference between means.

## Supplementary information

**Additional file 1 S1 File.** Analysis of variance (ANOVA) of microshoots number and length. Includes detailed and summary tables of ANOVA of treatment differences and Turkey comparison relatives to Table 2

**Additional file 2 S2 File.** Regenerated plants images underlying the results reported in the Fig. 1.

**Additional file 3 S3 File**. Analysis of variance (ANOVA) of microshoots root number and length. Includes detailed and summary tables of ANOVA of treatment differences and Turkey comparison relatives to Tables 3, 4, and 5.

**Additional file 4 S4 File.** Phytosanitary Certificate of Plant material. Includes detailed on the tractability.

**Additional file 5 S5 File.** Mother plants production under greenhouse. Includes detailed on cuttings planting.

**Additional file 6 S6 File.** Acclimatized plantlets underlying the results reported in the Fig. 7.

**Additional file 7 S1 Table.** Detailed data on sterilization experiment. Includes the average of clean explants.

**Additional file 8 S1 Raw.** images. Original uncropped images underlying the gel results reported in the Fig. 4.

**Additional file 9 S1 Raw-Data 1**. Original Data supporting Table 1.

**Additional file 10 S2 Raw-Data 2.** Original Data analysed to generate Table 2.

**Additional file 11 S3 Raw-Data 3.** Original Data analysed to generate Tables 3, 4, and 5.

## Data Availability

All data generated or analyzed during this study are included in this published article (and its supplementary information files).

## Abbreviations

CMD: Cassava mosaic disease
SSR: Simple sequence repeat
SCAR: Sequence-characterized amplified region
*CMD2*: Cassava mosaic disease resistant gene
CRD: Completely randomized design
MS: Murashige and skoog
TDZ: Thidiazuron
BAP: 6-benzylaminopurine
NAA: Naphtalen acetic acid
IBA: Indol butirivc acid
PAUSTI: Pan African university of basic sciences, technology and innovation
CRI: Coffee research institute

## References

[1] Burns A, Gleadow R, Cliff J, Zacarias A, Cavagnaro T (2010). Cassava the drought, war and famine crop in a changing world. Sustainability.

[2] Byrne D (1984). Breeding cassava. Plant Breed Rev.

[3] Guira F, Some K, Kabore D, Sawadogo-Lingani H, Traore Y, Savadogo A (2017). Origins, production, and utilization of cassava in Burkina Faso, a contribution of a neglected crop to household food security. Food Sci Nut.

[4] Houngue JA, Pita JS, Cacaï GHT, Zandjanakou-Tachin M, Abidjo EAE, Ahanhanzo C (2018). Survey of farmers’ knowledge of cassava mosaic disease and their preferences for cassava cultivars in three agro-ecological zones in Benin. J Ethnobiol Ethnomed.

[5] Agre AP, Gueye B, Adjatin A, Dansi M, Bathacharjee R, Rabbi IY, et al. Folk taxonomy and traditional management of cassava (*Manihot esculenta* Crantz) diversity in southern and Central Benin. Int J Inn Sci Res. 2016;20(2):500–15.

[6] Sessou AF, Kahia JW, Ateka E, Houngue JA, Dadjo C, Njenga P, Ahanhanzo C. Callus induction in three mosaic disease resistant cassava cultivars in Benin and genetic stability of the induced calli using simple sequence repeat (SSR) and sequence-characterized amplified region (SCAR) markers. Afr J Biot. 2019;18(31):1044–53.

[7] Bull SE, Ndunguru J, Gruissem W, Beeching JR, Vanderschuren H (2011). Cassava constraints to production and the transfer of biotechnology to African laboratories. Plant Cell Rep.

[8] Houngue JA, Zandjanakou-Tachin M, Ngalle HB, Pita JS, Cacaï GHT, Ngatat SE (2019). Evaluation of resistance to cassava mosaic disease in selected African cassava cultivars using combined molecular and greenhouse grafting tools. Phy Mol Plant Path.

[9] Fondong VN (2017). The search for resistance to cassava mosaic geminiviruses: how much we have accomplished, and what lies ahead. Frontiers.

[10] Rabbi IY, Hamblin MT, Kumar PL, Gedil MA, Ikpan AS, Jannink J-L (2014). High-resolution mapping of resistance to cassava mosaic geminiviruses in cassava using genotyping-by-sequencing and its implications for breeding. Virus Res.

[11] Cacaï GHT, Ahanhanzo C, Dangou JS, Houedjissin SS, Agbangla C (2012). Effets de différentes combinaisons hormonales Sur l’organogenèse in vitro de quelques cultivars locaux et variétés améliorées de Manihot esculenta Crantz (manioc-*Euphorbiaceae*) cultivées au Bénin. Int J Bio Chem Sci.

[12] Cacaï GHT, Adoukonou-Sagbadja H, Kumulugui BS, Ovono PO, Houngue J, Ahanhanzo C (2013). Eradication of cassava (Manihot esculenta) mosaic symptoms through thermotherapy and meristems cultured *in vitro*. Int J Agr Plant Pro.

[13] Oseni OM, Pande V, Nailwal TK (2018). A review on plant tissue culture, a technique for propagation and conservation of endangered plant species. Int J Curr Microbiol App Sci.

[14] Abd-Alla NA, Ragab ME, El-Miniawy SEM, Taha HS (2013). In vitro studies on cassava plant micropropagation of cassava (*Manihot esculenta* Crantz). J App Sci Res.

[15] Demeke Y, Tefera W, Dechassa N, Abebie B (2014). Effects of plant growth regulators on in vitro cultured nodal explants of cassava (*Manihot esculenta* Crantz) clones. Afr J Bio.

[16] Magaia HE (2015). Assessment and induction of variability through in vitro mutagenesis in cassava (*Manihot esculenta*, Crantz).

[17] Konan NK, Schöpke C, Carcamo R, Beachy RN, Fauquet C (1997). An efficient mass propagation system for cassava (*Manihot esculenta* Crantz) based on nodal explants and axillary bud-derived meristems. Plant Cell Rep.

[18] Mapayi EF, Ojo DK, Oduwaye OA, Porbeni JBO (2013). Optimization of *in-vitro* propagation of cassava (*Manihot esculenta* Crantz) genotypes. J Agr Sci.

[19] Murashige and Skoog (1962). A revised medium for rapid growth and bio assays with tobacco tissue cultures. Physiol Plant.

[20] Medina RD, Faloci MM, Gonzalez AM, Mroginski LA (2007). *In vitro* cultured primary roots derived from stem segments of cassava (*Manihot esculenta*) can behave like storage organs. Ann Bot.

[21] Acedo VZ, Labana CU. Rapid propagation of released Philippine cassava varieties through tissue culture. J Root Crops. 2008;34(2):108–14.

[22] Mushiyimana I, Hakizimana E, Gashaka G, Sallah PYK, Kalisa S, Gatunzi F (2011). Micro-propagation of disease resistant cassava variety in Rwanda. Rwanda J.

[23] Yandia SP, Gandonou CB, Silla S, Zinga I, Toukourou F (2018). Response of four cultivars of cassava (*Manihot esculenta* Crantz) plantlets free of cassava mosaic virus to micropropagation in different media. Afr J Biot.

[24] Bairu MW, Aremu AO, Van Staden J (2010). Somaclonal variation in plants: causes and detection methods. Plant Gro Reg.

[25] Beyene G, Chauhan RD, Wagaba H, Moll T, Alicai T, Miano D (2016). Loss of *CMD2*-mediated resistance to cassava mosaic disease in plants regenerated through somatic embryogenesis. Mol Plant Pat.

[26] Okogbenin E, Egesi CN, Olasanmi B, Ogundapo O, Kahya S, Hurtado P (2012). Molecular marker analysis and validation of resistance to cassava mosaic disease in elite cassava cultivars in Nigeria. Crop Sci.

[27] Mahungu NM, Dixon AGO, Kumbira JM. Breeding cassava for multiple pest resistance in Africa. Afr Crop Sci J. 1994;2(4):539–52.

[28] Maruthi MN, Whitfield EC, Otti G, Tumwegamire S, Kanju E, Legg JP (2019). A method for generating virus-free cassava plants to combat viral disease epidemics in Africa. Physiol Mol Plant Pathol.

[29] Waweru B, Ishimwe R, Kajuga J, Kagiraneza B, Sallah PYK, Ahishakiye V (2011). *In vitro* plant regeneration of cyphomandra betacea through nodal culture. Rwanda J.

[30] Abu-Romman SM, Al-Hadid KA, Arabiyyat AR (2015). Kinetin is the most effective cytokinin on shoot multiplication from cucumber. J Agri Sci.

[31] Garland P, Stoltz LP (1981). Micropropagation of Pissardi plum. Ann Bot.

[32] Huy NP, Luan VQ, Tung HT, Hien VT, Ngan HTM, Duy PN (2019). *In vitro* polyploid induction of *Paphiopedilum villosum* using colchicine. Sci Hort.

[33] Lane DW (1979). In vitro propagation of Spirea bumalda and Prunus cistena from shoot apices. Can J Plant Sci.

[34] Faye A, Sagna M, Kane PMD, Sane D (2015). Effects of different hormones on organogenesis in vitro of some varieties of cassava (*Manihot esculenta* Crantz) grown in Senegal. African J Plant Sci.

[35] Kabir MH, Mamun ANK, Roy PK, Islam MR, Jahan MT, Talukder SU (2015). *In vitro* propagation of cassava (*Manihot esculenta* Crantz). Nuclear Sci Appl.

[36] Opabode JT (2017). Enhanced mass regeneration of pro-vitamin a cassava (*Manihot esculenta* Crantz) varieties through multiple shoot induction from enlarged axillary buds. BioTechnologia.

[37] Sesay JV, Yamba NGG, Sherman-Kamara J, Quee DD (2018). Development of *in vitro* propagation protocol for some recalcitrant cassava (*Manihot esculenta* Crantz) genotypes in Sierra Leone. Afr J Biot.

[38] Martin AF, Ermayanti TM, Hapsari BW, Rantau DE. Rapid micropropagation of *Tacca leontopetaloides* (L.) Kuntze: The 5th Indonesia Biotechnology Conference; Hal. 2012. p. 240–551.

[39] Opabode JT, Ajibola OV, Akinyemiju OA (2015). Shoot induction from axillary bud of β-carotene enriched *Manihot esculenta* crantz and molecular stability of regenerants. Agric Trop Subtrop.

[40] Sukmadjaja D, Widhiastuti H (2011). Effects of plant growth regulators on shoot multiplication and root induction of cassava varieties culture *in vitro*. Southeast Asian J Trop Biol.

[41] Onwubiku IOI (2007). Micropropagation of cassava (*Manihot esculantum* Crantz) using different concentrations of benzyaminiopurine (BAP). J Eng Appl Sci.

[42] Kirika MW, Kahia JW, Diby LN, Njagi EM, Dadjo C, Kouame C (2015). Micropropagation of an endangered medicinal and indigenous multipurpose tree species. *Erythrina abyssinica*. Hort Sci.

[43] Shiji R, George J, Sunitha S, Muthuraj R (2015). Micropropagation for rapid multiplication of planting material in cassava (*Manihot esculenta* Crantz). J Root Crops.

[44] De Klerk G-J (2002). Rooting of microcuttings; theory and practice. In Vitro Cell Dev Bio-Plant.

[45] Naranjo C, Fallas E (2017). *Ex vitro* establishment and macropropagation of cassava (*Manihot esculenta*) to obtain disease-free rooted plants. VII Int Symp Prod Establishment Micropropagated Plants.

[46] Sadeghi F, Yadollahi A, Kermani MJ, Eftekhari M (2015). Optimizing culture media for in vitro proliferation and rooting of tetra (*Prunus empyrean* 3) rootstock. J Gen Eng Biot.

[47] Singh CK, Raj SR, Jaiswal PS, Patil VR, Punwar BS, Chavda JC (2016). Effect of plant growth regulators on *in vitro* plant regeneration of sandalwood (*Santalum album* L.) via organogenesis. Agrofor Syst.

[48] Bentz SE, Parliman BJ, Talbott H-J, Ackerman WL (1988). Factors affecting in vitro propagation of Yucca glauca. Plant Cell Tissue Org Cul.

[49] Vinterhalter B, Milošević DK, Janković T, Milojević J, Vinterhalter D (2012). *In vitro* propagation of *Gentiana dinarica* Beck. Cen Eur J Biol.

[50] Chauhan RD, Beyene G, Taylor NJ (2018). Multiple morphogenic culture systems cause loss of resistance to cassava mosaic disease. BMC Plant Biol.

[51] Diniz LEC, Ruas CF, Carvalho VP, Torres FM, Ruas EA, Santos MO (2005). Genetic diversity among forty coffee varieties assessed by RAPD markers associated with restriction digestion. Bra Arc Bio Tec.

[52] Omingo DO, Omondi CO, Cheserek J, Runo S, Okun D (2017). Diversity analysis of selected coffee genotypes using microsatellites and random amplified polymorphic DNA in Kenya. Intl J Biot Food Sci.

