# Supplementary figures and images for: In vitro propagation of three mosaic disease resistant cassava cultivars
Source: BMC Biotechnol. 2020 Sep 29;20:51. doi: 10.1186/s12896-020-00645-8 (PMC7526170; doi:10.1186/s12896-020-00645-8)

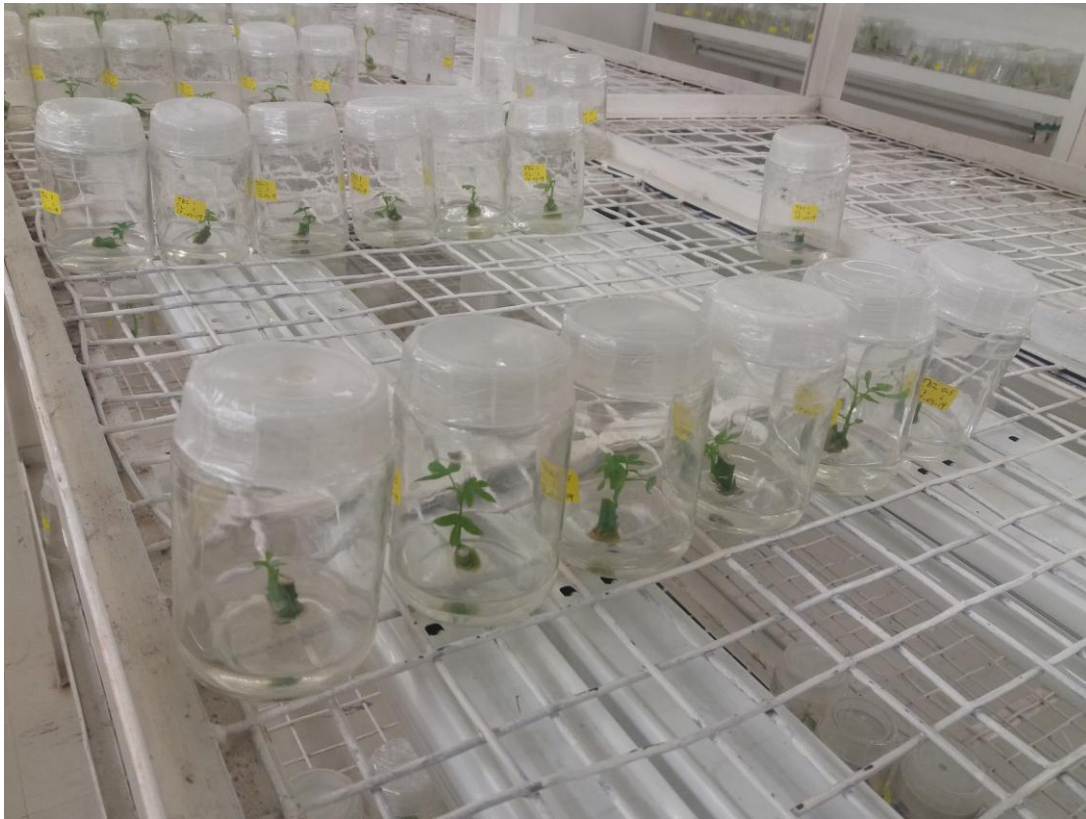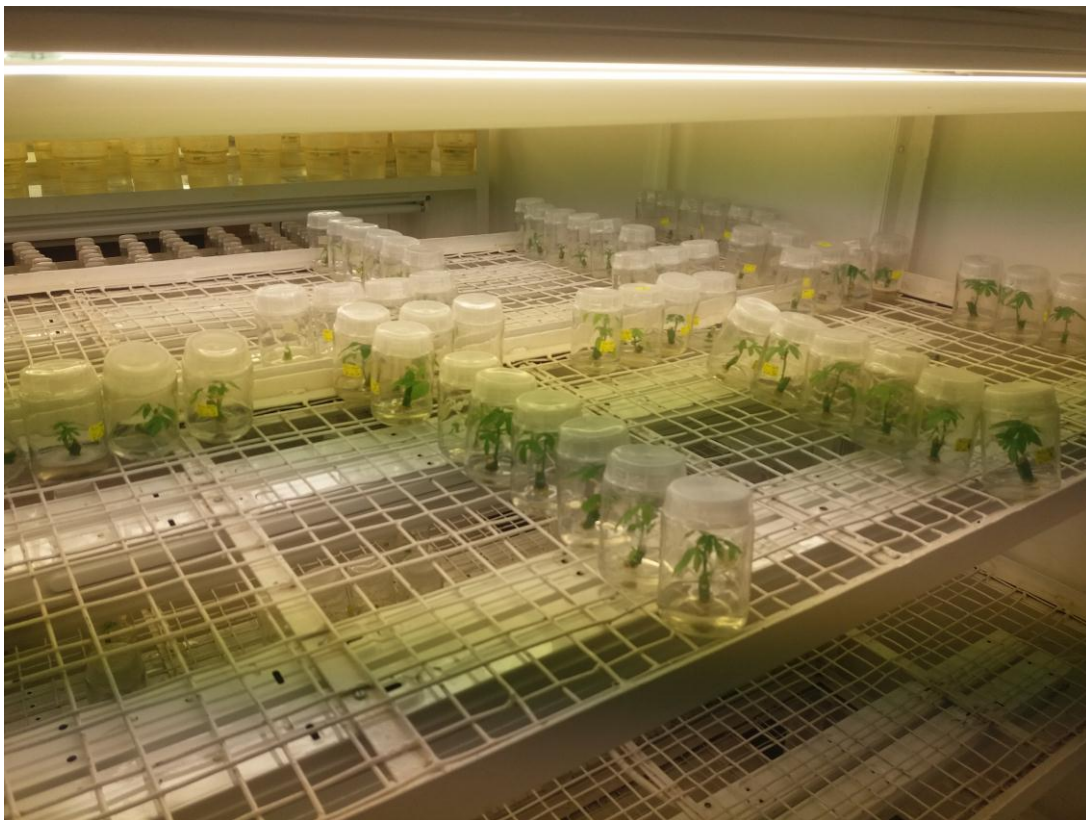

Supplement: Supplementary file 2 — Additional file 2 S2 File. Regenerated plants images underlying the results reported in the Fig. 1. [file 12896_2020_645_MOESM2_ESM.pdf]

Planted cuttings in the boats contain a soil

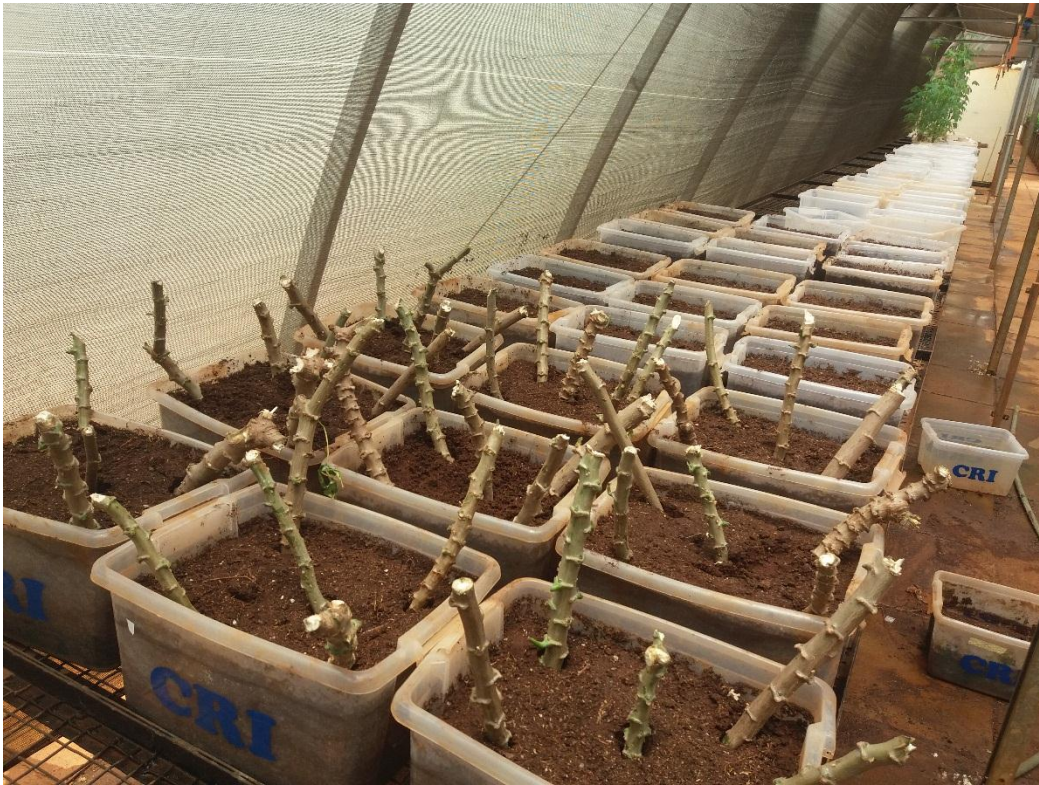

Cuttings sprouted two weeks after planting

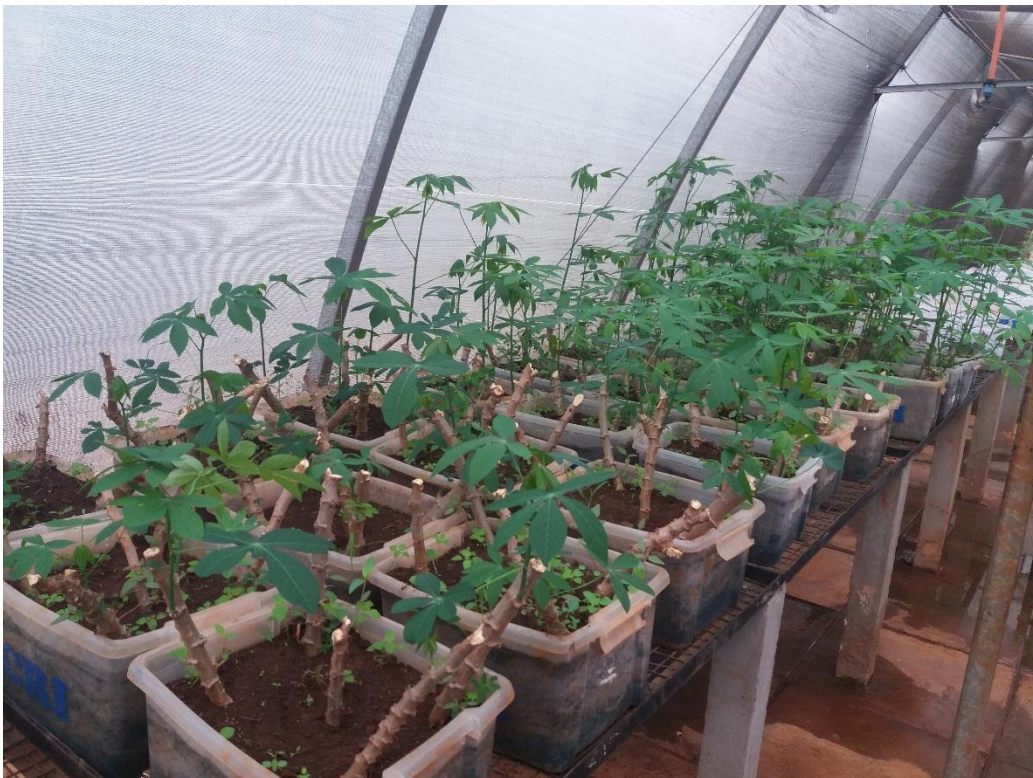

Supplement: Supplementary file 5 — Additional file 5 S5 File. Mother plants production under greenhouse. Includes detailed on cuttings planting. [file 12896_2020_645_MOESM5_ESM.pdf]

## Acclimatization of plantlets

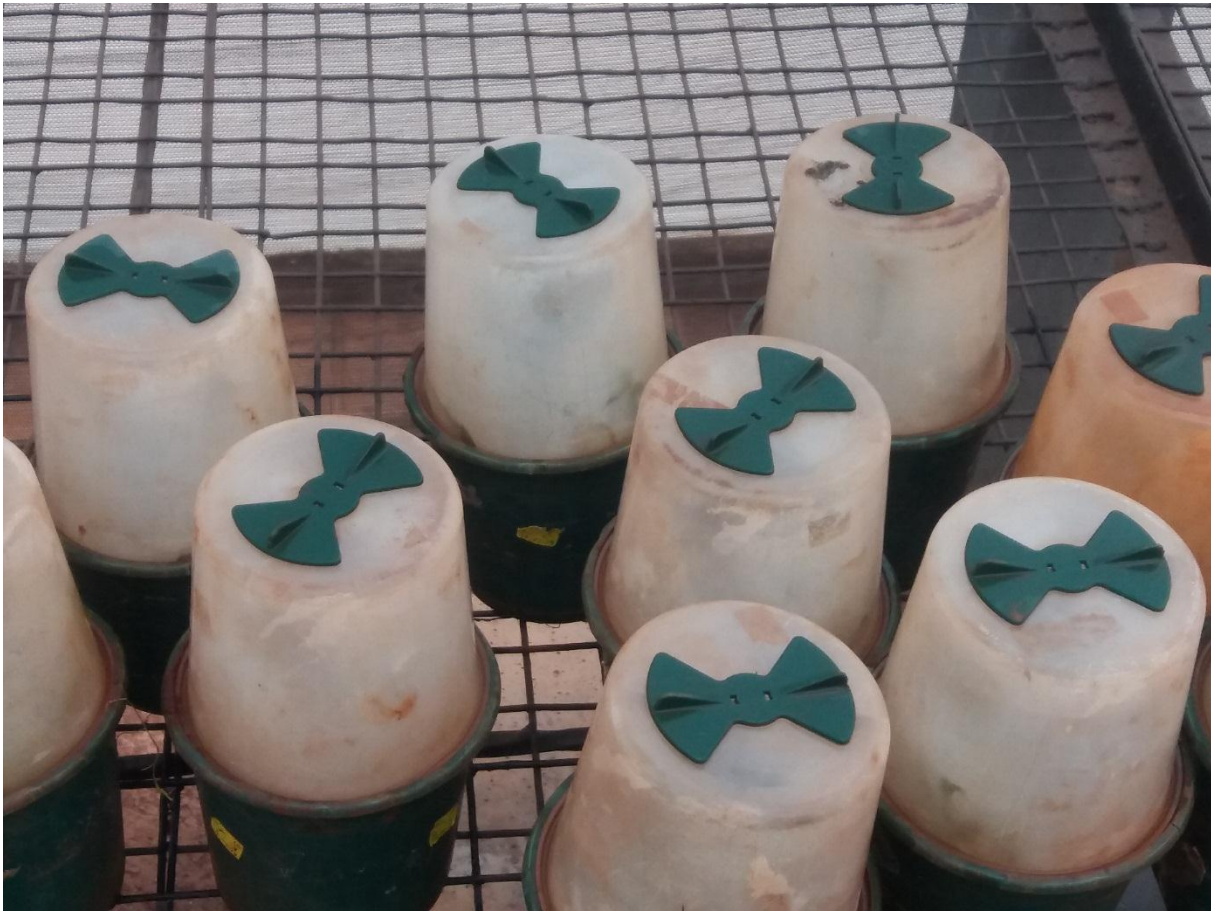

Supplement: Supplementary file 6 — Additional file 6 S6 File. Acclimatized plantlets underlying the results reported in the Fig. 7. [file 12896_2020_645_MOESM6_ESM.pdf]
