# Supplementary material for: In vitro propagation of three mosaic disease resistant cassava cultivars
Source: BMC Biotechnol. 2020 Sep 29;20:51. doi: 10.1186/s12896-020-00645-8 (PMC7526170; doi:10.1186/s12896-020-00645-8)
Supplement: Supplementary file 4 — Additional file 4 S4 File. Phytosanitary Certificate of Plant material. Includes detailed on the tractability. [file 12896_2020_645_MOESM4_ESM.pdf]

No. 1724846

KEPHIS CERT 1724846

PERMIT No. KEPHIS/21190/2018

REPUBLIC OF KENYA

MINISTRY OF AGRICULTURE & RURAL DEVELOPMENT

KENYA PLANT HEALTH INSPECTORATE SERVICE (KEPHIS)

PLANT IMPORTATION PERMIT

(Plant Protection Act Cap 324)

Date 9 October, 2018

The importer must furnish the supplier with a copy of this import permit before plant material is despatched.

*Permission is hereby granted to AMITCHIHOUE FRANK SESSOU, PAUSTI

of P.O. BOX 62000-00200, NAIROBI, KENYA

to import from JEROME HOUNGUE, UNIVERSITY OF ABOMEY-CALAVI

the following Stems

100 Stems Manihot esculenta

subject to the following conditions

1) All Stems to be the produce of and grown in BENIN
2) The consignment to be inspected on arrival and the importing authority reserves the right to treat, destroy or refuse the importation.
3) Plants or plantparts must be entirely free from soil, chaff and/or leaf mould.
4) Each consignment shall be accompanied by an original copy of this import permit and Phytosanitary Certificate (International Model or its equivalent) from country of origin;

Additional Declarations:

i) The Material must be used for Research purposes only
ii) Nature of research to be declared on request by KEPHIS inspector.
iii) To be used in approved quarantine facility.
Details to be stated on the Phytosanitary Certificate.

Failure to furnish the required certificates may result in prohibition of entry of the plant materials.

5) Packaging The following materials must **not** be used: banana leaves, maize, rice, sorghum, palm, wheat straw soil or leaf mould. If any other plant residue is used as packaging material, the consignment must be accompanied by a certificate stating: all seeds, pathogens and insects have been killed before use of the material either by heating to 180°F / 83°C for ten minutes or by chemical treatment (N.B:- Details to be stated on Phytosanitary Certificate).

This permit is valid for six months from date of issue, but may be cancelled at any time by the Director of Agriculture or by the officer issuing the permit on his behalf

Official Stamp

REGINA WACERA KABIRU

(Signed) [signature]

for Director of Agriculture

MINISTRY OF AGRICULTURE PLANT HEALTH INSPECTORATE SERVICE (NAIROBI) 09 OCT 2018 Sign... PLANT INSPECTOR P.O. Box ... NAIROBI KEPHIS

"Import of genetically modified material will require clearance from the National Biosafety Authority in compliance with the Biosafety Act"

The permission hereby granted is additional to any permission or licence required under any other law.
Full name and address of supplier to be stated

REPUBLIQUE DU BENIN

MINISTERE DE L'AGRICULTURE
DE L'ELEVAGE ET DE LA PECHE

DIRECTION DE LA PRODUCTION VEGETALE

1 **SERVICE DE LA PROTECTION DES VEGETAUX ET DU CONTROLE PHYTOSANITAIRE**

01 BP 58 Porto-Novo
Tél.: +229 20 21 32 90 / 20 21 32 93 - Fax : +229 20 21 44 13
spv.benin@yahoo.fr

2 **CERTIFICAT PHYTOSANITAIRE**
N° 0054994/19/SPVCP/PCP-AE/B

1 Le Service de la Protection des Végétaux et du Contrôle Phytosanitaire du Bénin

Au
Service de la Protection des Végétaux de KENYA.

3 Lieu d'origine BENIN.

4 Nom et adresse de l'expéditeur<br>SESSOU A. FRANCK.<br>TEL: 95906976	5 Nom et adresse du destinataire<br>SESSOU A. FRANCK.<br>JUJA KENYA
6 Moyen de transport déclaré	7 Point d'entrée déclaré JUJA.
8 Marques des colis: nombre et nature des colis : nom commun du produit/organisme animal : nom scientifique des plantes/organismes animaux<br>0 COLIS D'échantillon de produit Agricole:<br>- Boutures de: Manihot esculenta.	9 Quantité déclarée<br>07kg.

10 Il est certifié que les végétaux ou produits végétaux ou organismes animaux décrits ci-dessus
- ont été inspectés suivant des procédures adaptées, et
- estimés indemnes d'ennemis visés par la réglementation et pratiquement indemnes d'autres ennemis dangereux, et
- sont jugés conformes à la réglementation phytosanitaire en vigueur dans le pays importateur.

Timbre fiscal

11 Déclaration supplémentaire
ECHANTILLON (SAMPLE).

TRAITEMENT DE DESINFECTATION ET/OU DE DESINFECTION

12 Traitement<br>NEANT		18 Lieu de délivrance Cotonou<br>19 Date Le 09/03/19
13 Produit chimique (matière active)<br>NEANT	14 Durée et température<br>NEANT	20 Nom et signature du Fonctionnaire autorisé<br>[signature]
15 Concentration<br>NEANT	16 Date<br>NEANT	21 Cachet de l'organisation<br>REPUBLIQUE DU BENIN * POSTE DE CONTRÔLE PHYTOSANITAIRE DE L'AEROPORT 4 * MAEP * DPV * SPVCP
17 Renseignements complémentaires<br>NEANT.		

<u>NB</u> : *Le certificat phytosanitaire n'est délivré que sur la base d'un contrôle de la marchandise à exporter en tenant compte des exigences phytosanitaires du pays importateur. Il est subordonné à l'autorisation de l'importation attestée par le Permis d'importation du pays importateur.*
