# Supplementary material for: In vitro propagation of three mosaic disease resistant cassava cultivars
Source: BMC Biotechnol. 2020 Sep 29;20:51. doi: 10.1186/s12896-020-00645-8 (PMC7526170; doi:10.1186/s12896-020-00645-8)
Supplement: Supplementary file 7 — Additional file 7 S1 Table. Detailed data on sterilization experiment. Includes the average of clean explants. [file 12896_2020_645_MOESM7_ESM.docx]

| **Cultivars** | **Number of Explants** | | **Jik Concentration (%)** | **Exposure time (min)** | **After 4 days**  **Table:** Details data on sterilization experiment | | | **After 8 days** | | | **After 12 days** | | |
| --- | --- | --- | --- | --- | --- | --- | --- | --- | --- | --- | --- | --- | --- |
|  |  |  |  |  | Nb. of clean explants | (%) Clean explants/cultivars | (%) Clean explants/treatement | Nb. of clean explants | (%) Clean explants/cultivars | (%) Clean explants/treatement | Nb. of clean explants | (%) Clean explants/cultivars | (%) Clean explants/treatement |
| Agblehoundo | | 20 | **10** | **5** | 17 | 85 | **80** | 15 | 75 | **66.667** | 2 | 10 | **13.333** |
| Agric-rouge | | 20 |  |  | 16 | 80 |  | 13 | 65 |  | 3 | 15 |  |
| Atinwewe | | 20 |  |  | 15 | 75 |  | 12 | 60 |  | 3 | 15 |  |
| Agblehoundo | | 20 | **10** | **10** | 16 | 80 | **81.667** | 14 | 70 | **63.333** | 2 | 10 | **13.333** |
| Agric-rouge | | 20 |  |  | 14 | 70 |  | 11 | 55 |  | 2 | 10 |  |
| Atinwewe | | 20 |  |  | 19 | 95 |  | 13 | 65 |  | 4 | 20 |  |
| Agblehoundo | | 20 | **10** | **15** | 17 | 85 | **78.333** | 4 | 20 | **21.667** | 3 | 15 | **13.333** |
| Agric-rouge | | 20 |  |  | 15 | 75 |  | 5 | 25 |  | 2 | 10 |  |
| Atinwewe | | 20 |  |  | 15 | 75 |  | 4 | 20 |  | 3 | 15 |  |
| Agblehoundo | | 20 | **10** | **20** | 19 | 95 | **90** | 15 | 75 | **78.333** | 11 | 55 | **55** |
| Agric-rouge | | 20 |  |  | 19 | 95 |  | 15 | 75 |  | 12 | 60 |  |
| Atinwewe | | 20 |  |  | 16 | 80 |  | 17 | 85 |  | 10 | 50 |  |
| Agblehoundo | | 20 | **15** | **5** | 19 | 95 | **91.667** | 14 | 70 | **56.667** | 10 | 50 | **43.333** |
| Agric-rouge | | 20 |  |  | 18 | 90 |  | 10 | 50 |  | 9 | 45 |  |
| Atinwewe | | 20 |  |  | 18 | 90 |  | 10 | 50 |  | 7 | 35 |  |
| Agblehoundo | | 20 | **15** | **10** | 19 | 95 | **93.333** | 13 | 65 | **21.667** | 7 | 35 | **28.333** |
| Agric-rouge | | 20 |  |  | 19 | 95 |  | 14 |  |  | 5 | 25 |  |
| Atinwewe | | 20 |  |  | 18 | 90 |  | 16 |  |  | 5 | 25 |  |
| Agblehoundo | | 20 | **15** | **15** | 20 | 100 | **100** | 20 | 100 | **100** | 15 | 75 | **73.333** |
| Agric-rouge | | 20 |  |  | 20 | 100 |  | 20 | 100 |  | 15 | 75 |  |
| Atinwewe | | 20 |  |  | 20 | 100 |  | 20 | 100 |  | 14 | 70 |  |
| Agblehoundo | | 20 | **15** | **20** | 20 | 100 | **100** | 15 | 75 | **71.667** | 14 | 70 | **71.667** |
| Agric-rouge | | 20 |  |  | 20 | 100 |  | 12 | 60 |  | 15 | 75 |  |
| Atinwewe | | 20 |  |  | 20 | 100 |  | 16 | 80 |  | 14 | 70 |  |
| Agblehoundo | | 20 | **20** | **15** | 20 | 100 | **100** | 20 | 100 | **96.667** | 19 | 95 | **90** |
| Agric-rouge | | 20 |  |  | 20 | 100 |  | 19 | 95 |  | 16 | 80 |  |
| Atinwewe | | 20 |  |  | 20 | 100 |  | 19 | 95 |  | 19 | 95 |  |
| Agblehoundo | | 20 | **20** | **20** | 19 | 95 | **93.333** | 17 | 85 | **91.667** | 17 | 85 | **81.667** |
| Agric-rouge | | 20 |  |  | 20 | 100 |  | 20 | 100 |  | 15 | 75 |  |
| Atinwewe | | 20 |  |  | 17 | 85 |  | 18 | 90 |  | 17 | 85 |  |
| Agblehoundo | | 20 | **25** | **15** | 19 | 95 | **95** | 19 | 95 | **80** | 16 | 80 | **80** |
| Agric-rouge | | 20 |  |  | 20 | 100 |  | 15 | 75 |  | 16 | 80 |  |
| Atinwewe | | 20 |  |  | 18 | 90 |  | 14 | 70 |  | 16 | 80 |  |
| Agblehoundo | | 20 | **25** | **20** | 20 | 100 | **96.667** | 20 | 100 | **91.667** | 17 | 85 | **85** |
| Agric-rouge | | 20 |  |  | 19 | 95 |  | 17 | 85 |  | 19 | 95 |  |
| Atinwewe | | 20 |  |  | 19 | 95 |  | 18 | 90 |  | 15 | 75 |  |
