# Supplementary material for: In vitro propagation of three mosaic disease resistant cassava cultivars
Source: BMC Biotechnol. 2020 Sep 29;20:51. doi: 10.1186/s12896-020-00645-8 (PMC7526170; doi:10.1186/s12896-020-00645-8)

C1 = mother plants of Agric-rouge; R1 = Regenerated plants of Agric-rouge; C2 = mother plants of Atinwewe; R2 = Regenerated plants of Atinwewe; C3 = mother plants of Agblehoundo; R3= Regenerated plants of Agblehoundo.

SCARs amplification of CMD2 in Cassava

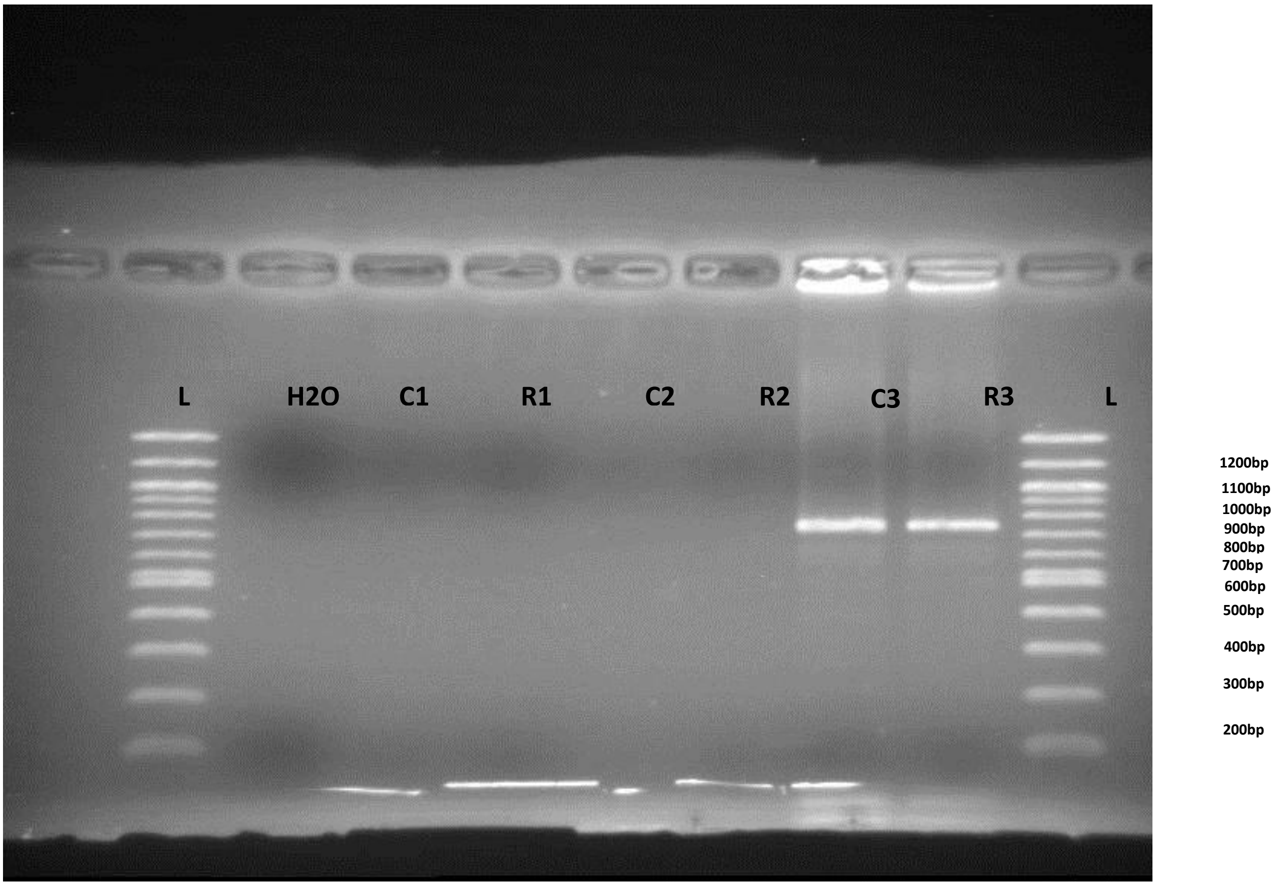

SSRs amplification of CMD2 in cassava

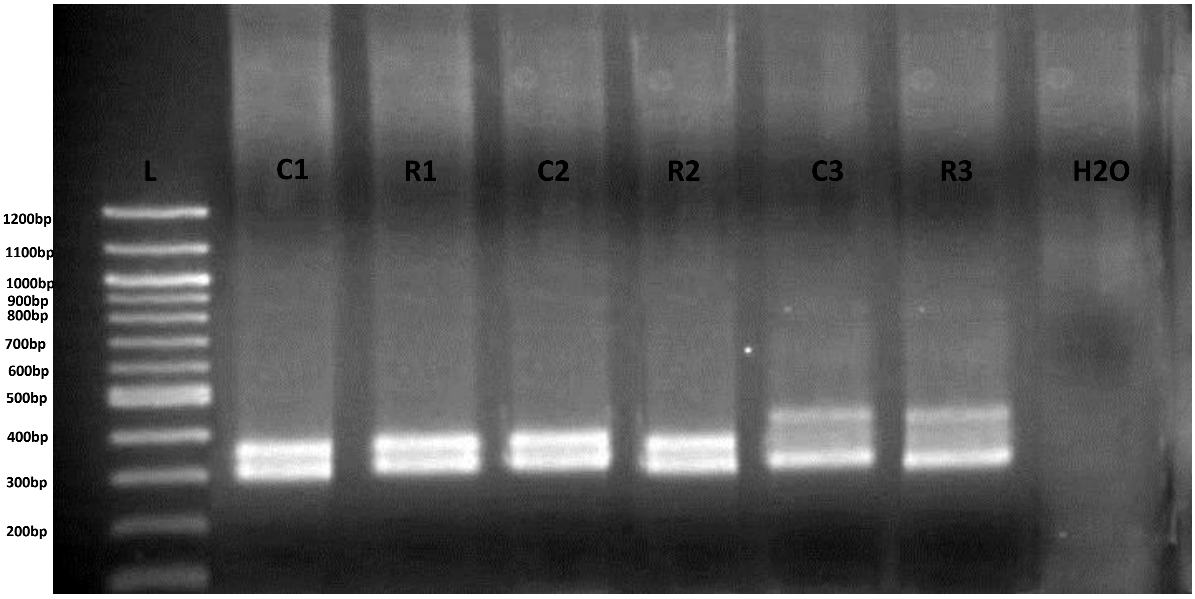

Supplement: Supplementary file 8 — Additional file 8 S1 Raw. images. Original uncropped images underlying the gel results reported in the Fig. 4. [file 12896_2020_645_MOESM8_ESM.pdf]
